# SAR and QSAR modeling of a large collection of LD_50_ rat acute oral toxicity data

**DOI:** 10.1186/s13321-019-0383-2

**Published:** 2019-08-30

**Authors:** Domenico Gadaleta, Kristijan Vuković, Cosimo Toma, Giovanna J. Lavado, Agnes L. Karmaus, Kamel Mansouri, Nicole C. Kleinstreuer, Emilio Benfenati, Alessandra Roncaglioni

**Affiliations:** 1 Laboratory of Environmental Chemistry and Toxicology, Department of Environmental Health Sciences, Istituto di Ricerche Farmacologiche Mario Negri IRCCS, Via Mario Negri 2, 20156 Milan, Italy; 2 Integrated Laboratory Systems, Research Triangle Park, NC 27560 USA; 3 NTP Interagency Center for the Evaluation of Alternative Toxicological Methods, National Institute of Environmental Health Sciences, Research Triangle Park, NC 27560 USA; 4 Institute for Risk Assessment Sciences, Utrecht University, PO Box 80177, 3508 TD Utrecht, The Netherlands

**Keywords:** (Q)SAR, Acute rat oral toxicity, LD50, Integrated modeling, Computational toxicology

## Abstract

The median lethal dose for rodent oral acute toxicity (LD50) is a standard piece of information required to categorize chemicals in terms of the potential hazard posed to human health after acute exposure. The exclusive use of in vivo testing is limited by the time and costs required for performing experiments and by the need to sacrifice a number of animals. (Quantitative) structure–activity relationships [(Q)SAR] proved a valid alternative to reduce and assist in vivo assays for assessing acute toxicological hazard. In the framework of a new international collaborative project, the NTP Interagency Center for the Evaluation of Alternative Toxicological Methods and the U.S. Environmental Protection Agency’s National Center for Computational Toxicology compiled a large database of rat acute oral LD50 data, with the aim of supporting the development of new computational models for predicting five regulatory relevant acute toxicity endpoints. In this article, a series of regression and classification computational models were developed by employing different statistical and knowledge-based methodologies. External validation was performed to demonstrate the real-life predictability of models. Integrated modeling was then applied to improve performance of single models. Statistical results confirmed the relevance of developed models in regulatory frameworks, and confirmed the effectiveness of integrated modeling. The best integrated strategies reached RMSEs lower than 0.50 and the best classification models reached balanced accuracies over 0.70 for multi-class and over 0.80 for binary endpoints. Computed predictions will be hosted on the EPA’s Chemistry Dashboard and made freely available to the scientific community.

## Introduction

Over the past 25 years, synthetic organic chemical production world-wide has increased dramatically, from about 50 million tons to approximately 150 million tons [1]. This ever-growing increase of chemical substances represents a primary issue for the environment and human safety. Toxicological tests need to be performed to evaluate which of these chemicals are safe and which can potentially contaminate the environment and cause toxicity.

In the first stages of toxicological testing programs, acute toxicity studies are frequently used to categorize the agent in terms of the potential hazard posed to human health. Acute toxicity describes the adverse toxicological effects of a chemical that occur either from a single exposure or from multiple exposures in a short period of time (usually less than 24 h) [2].

The median lethal dose (LD50) is the basis for the toxicological classification of chemicals for various regulations concerning chemical hazard [3, 4]. The acute LD50 is the lethal dose of a substance that will kill 50% of the test animals/organisms within 24 h of exposure to the test substance [5–7]. Acute toxicity studies are conducted following various routes of exposure (e.g. oral, dermal and inhalation), and rodents are the most common animal model employed to estimate the lethal dose [8]. The estimation of rodent acute toxicity provides a baseline value when detailed toxicity data are unavailable for the chemical(s) of interest. In this case, LD50 values may be employed to make a first assessment of relative toxicity among chemicals [6].

However, the exclusive use of in vivo testing has obvious limitations, related to the high monetary and time cost of performing such experiments, the need to sacrifice a number of animals, and the number of chemicals requiring assessment. Indeed, it has been reported that toxicological and other safety evaluations represent 8% of the total number of animals used for experimental purposes in Europe, with rodents being the most commonly used specie [9, 10].

In light of this, recent laws are pushing the acceptance of alternative methods (e.g., in vitro and in silico methods) and their use by the regulatory and public health bodies in order to reduce the use of animals [11, 12]. Computational toxicology is a viable approach to reduce both the cost and the number of animals used for experimental toxicity assessment [13].

Structure–activity relationship and quantitative structure–activity relationship [(Q)SAR] models are in silico approaches to determine the toxicity of a large number of chemicals by analyzing their chemical structure. These methods are increasingly used to fill the toxicological data gaps for high-production volume chemicals (e.g., pharmaceutical, agrochemical, food additives and industrial) [3, 6, 14, 15] because they require a relatively small amount of resources and time.

Despite this, the reported number of (Q)SAR studies on mammalian toxicity is limited [3, [9], with the majority being restricted to particular classes of chemicals and based on small, focused datasets [16, 17, 18].

Recently, the NTP Interagency Center for the Evaluation of Alternative Toxicological Methods (NICEATM) in collaboration with the U.S. Environmental Protection Agency (EPA) National Center for Computational Toxicology (NCCT) compiled a large list of rat acute oral LD50 data on ~ 12 k chemicals. These data have been made available to the scientific community, to serve as the basis for an international collaborative modeling initiative. The modeling initiative was launched by the Interagency Coordinating Committee on the Validation of Alternative Methods (ICCVAM) Acute Toxicity Workgroup with the aim to develop new computational models for predicting five specific acute oral systemic toxicity endpoints required for regulatory purposes [19].

These five endpoints of regulatory significance for acute oral toxicity included the identification of (1) “very toxic” chemicals (i.e., LD50 less than 50 mg/kg); (2) “non-toxic” chemicals (LD50 greater than or equal to 2000 mg/kg); (3) point estimates for rodent LD50; (4) categorization of toxicity hazard using the U.S. EPA classification scheme [20]; (5) categorization of toxicity hazard using the United Nations Globally Harmonized System of Classification and Labelling (GHS) classification schemes (United Nations 2009).

The present article encompasses all the efforts that our research group contributed to this initiative. Both regression and classification computational models were developed for the five endpoints, by employing several statistical (i.e. QSAR) and knowledge-based (i.e. SAR) methods. External validation performance was provided to demonstrate the predictive capacity of the models. In the end, integrated modeling strategies were proposed to improve performance of single models. A multi-objective optimization based on the concept of Pareto optimum was proposed for identifying the best solution (i.e., individual or integrated model) for each modeled endpoint [21, 22].

## Methods

### Endpoints

The following endpoints were considered for modeling:

- LD50 single point estimates (continuous) expressed in mg/kg_bw_, and converted for modeling purposes in logarithm of mmol/kg_bw_.
- “Very toxic” (vT) binary classification: LD50 < 50 mg/kg (positive class) and LD50 ≥ 50 mg/kg (negative class).
- “Non-toxic” (nT) binary classification: LD50 > 2000 mg/kg (positive class) and LD50 ≤ 2000 mg/kg (negative class)
- EPA’s 4-category hazard classification [20]:
  - Category I (LD50 ≤ 50 mg/kg) is the highest toxicity category. Category II (moderately toxic) includes chemicals with 50 < LD50 ≤ 500 mg/kg. Category III (slightly toxic) includes chemicals with 500 < LD50 ≤ 5000 mg/kg. Safe chemicals (LD50 > 5000 mg/kg) are included in Category IV.
- GHS 5-category hazard classification [23]:
  - Category I includes chemicals with LD50 ≤ 5 mg/kg. Category II includes chemicals with 5 < LD50 ≤ 50 mg/kg. Category III includes chemicals with 50 < LD50 ≤ 300 mg/kg. Category IV includes chemicals with 300 < LD50 ≤ 2000 mg/kg). Category V includes chemicals with LD50 > 2000 mg/kg.

### Datasets

NICEATM and NCCT compiled and curated a rat acute oral systemic toxicity (LD50) inventory with values expressed as mg/kg of body weight (bw). This dataset was split semi-randomly, i.e. ensuring equivalent coverage with respect to LD50 distribution (and corresponding classes and categories for binary and categorical endpoints), by the organizers of the project into a list of compounds to be used for modeling (75%; 8994 chemicals) and validation (i.e., evaluation set, ES) (25%; 2895 chemicals). All the data and project information were made available to the cheminformatics community by NICEATM and NCCT at https://ntp.niehs.nih.gov/pubhealth/evalatm/test-method-evaluations/acute-systemic-tox/models/index.html.

Project organizers retrieved acute toxicity data from several sources, i.e. Helmholtz Center for Environmental Research’s ChemProp (https://www.ufz.de/index.php?en=34601), the Joint Research Center’s Acutoxbase [24], the National Library of Medicine’s (NLM) Hazardous Substance Data Bank (HSDB) [25], the OECD’s eChemPortal (https://www.echemportal.org/echemportal/index.action), NICEATM’s Pesticide Active Ingredients (PAI) (https://ntp.niehs.nih.gov/pubhealth/evalatm/index.html) and NLM’s ChemIDPlus (https://chem.nlm.nih.gov/chemidplus/). Over the 75% of chemical structures were retrieved from the EPA’s DSSTox database [26], while the remaining were crosschecked from literature publications. Details on the preparation of these data and the related variability is discussed in a separate paper by Kleinstreuer et al. (in preparation). In this work, the list of 8994 chemicals proposed by NICEATM and NCCT for modeling was processed to create a training set (TS) for the development of models here presented, specific for each endpoint modeled. Project information reports the presence of 158 duplicate (Q)SAR-ready structures, mostly due to the presence of different counterions associated to the main molecule (https://ntp.niehs.nih.gov/iccvam/methods/acutetox/model/qna.pdf). Because (Q)SAR approaches applied in this work do not deal with counterions, duplicated entries were aggregated. A single experimental value was assigned to each unique chemical structure (see Table 1 for details on the composition of the TSs). Details on the procedure applied to obtain the final version of datasets is included in Additional file 1.

**Table 1:** Number of chemicals included in the TS and the ES for each toxicity class

	Training set (TS)						Evaluation set (ES)					
	#	Class 1	Class 2	Class 3	Class 4	Class 5	#	Class 1	Class 2	Class 3	Class 4	Class 5
LD50	6279	–	–	–	–	–	2169	–	–	–	–	–
vT	8462	703	7759	–	–	–	2888	242	2646	–	–	–
nT	8402	4779	3623	–	–	–	2884	1651	1233	–	–	–
EPA	8259	703	1806	4142	1608	–	2859	242	643	1421	553	–
GHS	8331	165	538	1089	2916	3623	2879	58	184	391	1013	1233

Hazard categories for the two multi-class modeled endpoints (EPA and GHS classes) are sorted for decreasing toxicity, from 1 to 5. For the vT classification, class 1 corresponds to the positive (i.e., very toxic) compounds, while for the nT endpoint class 1 corresponds to the negative (i.e., toxic) compounds

The ES was initially imbedded into a larger prediction set by NICEATM and NCCT to facilitate a blind evaluation of all the models that were developed by the various institutions during the initiative. It was subsequently released with all the information relative to the five endpoints (https://ntp.niehs.nih.gov/iccvam/methods/acutetox/model/validationset.txt). While the TS used here were a result of a reworking of data provided for modeling, the ES was used as released for validating models presented here. Deduplication was performed by organizers of the project based only on CAS registration numbers. Consequently, a small degree of superimposition was observed between the TS and the ES due to the presence of different CAS numbers pointing to the same chemical structure. This overlap in chemistry is limited (i.e., about 8% of ES chemicals were also included in the TS) and it does not undermine statistical relevance of validation results. Table 1 summarizes, for each endpoint, the number of chemicals included in each toxicity category for TS and ES. In all cases, TS and ES showed a nearly analogous distribution of chemicals among the various classes.

The entire TS and ES are included in Additional file 2: Table S1 and Additional file 2: Table S2. The TS was analyzed by means of a principal component analysis based on CDK descriptors [27] implemented in KNIME analytical platform [31]. Eight chemicals identified as structural outliers based on score values on the first two principal components were removed from the TS.

### Models development

Table 2 lists all the statistical methods applied for the development of models submitted to NICEATM for predicting rat oral acute toxicity. For each method, modelled endpoints were also specified. Below, technical details of each method are described.

**Table 2:** Summary of modeling methods used

Method	Software	Descriptors	Applicability domain	Endpoints				
				LD50 point estimate	vT	nT	EPA	GHS
BRF/rRF	KNIME	Dragon	“Error” model Confidence Similarity	✓	✓	✓	✓	✓
aiQSAR	R	Dragon	ADM	✓	✓	✓	✓	✓
istKNN	istKNN	Fingerprints + structural keys	Similarity/activity-based thresholds	✓				
SARpy	SARpy	SAs	Presence/absence of SAs		✓	✓		
HPT-RF	R (Caret)	Dragon	Mirror matrix Isolation forest	✓			✓	✓
GLM	R (H_2_O)	Dragon	NA			✓		

For each method, the software, the descriptors used, the applicability domain definition and the modeled endpoints are specified. The methods listed are balanced random forest (BRF)/regression random forest (rRF); ab initio QSAR (aiQSAR); istKNN; hyper-parameter tuning random forest (HPT-RF); generalized linear model (GLM)

#### Balanced random forest (BRF)/random forest in regression (rRF)

*Data split* For each compound, structural fingerprints were calculated using the Indigo toolkit implemented in KNIME (http://lifescience.opensource.epam.com/indigo/). K-means clustering was applied, taking into account the structural information (codified by the fingerprints) and the experimental values of each chemical. TS was split into an internal training set (iTS) (80%) and an internal validation set (iVS) (20%) based on a stratified sampling of the obtained clusters, to ensure structural and activity analogy between the two datasets. The iTS was used for the development of QSAR models, while the iVS was used for the tuning of model and applicability domain (AD) parameters. The number of chemicals included in iTS and iVS for each modeling endpoint is reported (“Results” section).

*Algorithms* Random Forest in regression (rRF) [28, 29] was applied for the derivation of LD50 single point estimate prediction models. For the categorical ones (i.e., nT, vT, GHS and EPA), balanced random forest (BRF) was used. This technique is a combination of under-sampling and the ensemble idea that artificially alters the class distribution so that classes are represented equally in each tree [30]. This allowed handling of unbalanced distributions of chemicals among classes for some of the categorical endpoints. The number of trees for each model was varied among 50, 100 and 150, then the best solution was selected based on performance on the iVS. All algorithms were implemented in the KNIME platform [31].

*Descriptors* Molecular descriptors were calculated for each compound using Dragon software [32]. Descriptors for iTS compounds were pruned by constant and semi-constant values (i.e., standard deviation < 0.01), then those having at least one missing value were removed. In case of highly correlated pairs of descriptors (i.e., absolute pair correlation higher than 95%), only one was retained and the descriptor showing the highest pair correlation with all the other descriptors was removed. Descriptors were normalized in the range of 0–1, then the same normalization scheme was applied to iVS descriptors.

*Applicability domain* Three approaches were applied for the definition of AD.

- *Similarity* A matrix containing pairwise Manhattan distances (based on Dragon descriptors used in the model) was calculated for iTS compounds. Chemicals were sorted based on the mean distance with respect to their first *k* neighbors and then the value corresponding to a given percentile of the distribution of distances was used as a threshold (T_D_). Chemicals with mean distances above T_D_ were excluded from the AD. The same procedure was repeated for iVS chemicals with respect to their neighbors in the iTS, for identifying compounds outside of AD. Values assigned to *k* were 1 and 5; values assigned to T_D_ were those corresponding to the 100th, the 97.5th, the 95th and the 90th percentiles of the iTS distance distribution. This method was applied on both continuous (LD50) and classification models.
- *Error model* An “error model” predicts the uncertainty of the predictions coming from a classical “activity model”. An error model was derived from the same iTS of the associated activity model, with the difference that the cross-validated absolute errors (previously generated by the activity model) represent the dependent variables while independent variables are represented by a series of AD metrics that reflect the accuracy of the predictions made by the activity model [33]. The RF algorithm was used for the error model derivation. iTS chemicals were sorted based on errors in prediction estimated by the error model, then the value corresponding to a given percentile of the distribution of predicted errors was used as a threshold (T_E_). Chemicals exceeding T_E_ were excluded from the AD. The same T_E_ was applied on predicted errors calculated for iVS chemicals. For the present work, values assigned to T_E_ corresponded to the 100th, the 90th, the 75th and the 65th percentile of the iTS errors distribution. This method was applied only on the continuous LD50 point estimate models.
- *Confidence* The percentage of trees within the RF yielding the same prediction (i.e., confidence) was estimated. This method was applied only for classification models. For binary classification models (i.e., vT and nT) a confidence threshold (T_C_) was gradually incremented by 0.05, from a minimum of 0.60 to a maximum of 0.75. For multi-class models (i.e., EPA and GHS), the confidence threshold was incremented by 0.10 from a minimum of 0.30 to a maximum of 0.70. Chemicals having confidence lower than this threshold were considered outside of the model AD.

For each model, the various parameters were evaluated in each possible combination, i.e. T_D_, *k*, T_E_ for continuous models, T_D_, *k*, T_C_ for classification models. The best combination of parameters was selected based on the best trade-off in terms of coverage and performance on the iVS (see “Results” section).

#### aiQSAR

*Data split* The entire TS was used to derive aiQSAR models.

**Fig. 1:**
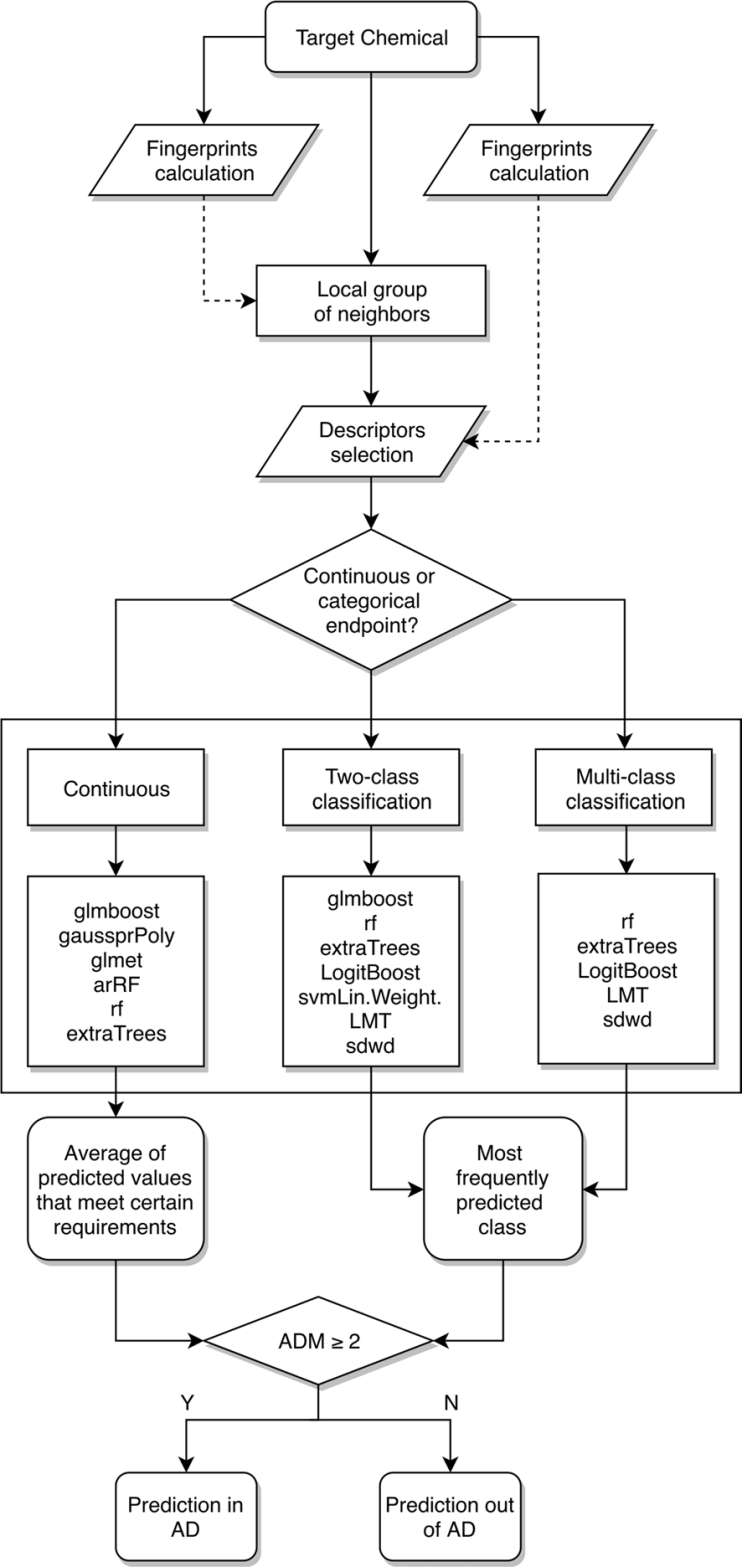
Workflow for aiQSAR-based model development (Adapted from [34])

*Algorithm* aiQSAR methodology described by Vukovic et al. [34] was applied for development of regression and classification models. This method is based on the-runtime derivation of local models specific to each compound (in this section referred to as the target compound). Each model is derived from a small local group of structurally similar compounds included in the TS. Figure 1 summarizes the entire aiQSAR workflow.

Similar compounds were selected on the basis of Tanimoto distances computed from the comparison of “PubChem” (ftp://ftp.ncbi.nlm.nih.gov/pubchem/specifications/pubchem_fingerprints.txt) and “Extended” fingerprints [35] between the target compound and all the TS compounds, as implemented in the “rcdk” R package [36]. A minimum of 20 and a maximum of 50 compounds were selected for the development of each model. First all compounds that are above the threshold value are selected (PubChem” similarity ≥ 0.80 and “Extended” similarity ≥ 0.70) Then, in case where the required number is not met, average ranks of compounds are considered to either add additional compounds, or to discard some of the selected ones.

Several mathematical models implemented in “caret” R package [37] were built from the local group of neighbors and each model was used to predict the value of the target compound. The type and the number of models varied based on the endpoint (i.e., regression, binary classification, multi-class classification) and is listed in Fig. 1. Further details on the methods used are reported in Vukovic et al. [34].

Finally, predictions from all methods were combined into a single output value for each target compound. For regression endpoints (i.e., LD50 point estimates), the final prediction was the average of predictions from individual methods, after discarding those more than 10% out of the range of experimental values of the TS. For classification endpoints (i.e., vT, nT, EPA and GHS), a majority vote approach was applied. In case of any tied votes, the class that is least represented in TS (out of the tied classes) was selected. In the present work, this always corresponded to the most toxic class in the tie being selected.

*Descriptors* Molecular descriptors were calculated using Dragon 7 software [32]. All available 1D and 2D descriptors (3839 overall) were considered for modeling purposes. Dragon descriptors with a missing value in any compound from the local group or in the target compound were iteratively discarded before each local model generation, as well as descriptors that were constant and near-constant within the local group of neighbors.

*Applicability domain* Applicability domain measure (ADM) of the target compound was computed based on average values of “PubChem” and “Extended” fingerprint similarities between the target compound and its local group of neighbors. The more similar is the local group of neighbors, the higher is the ADM score, that ranges between 1 (out of AD) and 5 (in AD). For the present work, chemicals with ADM ≥ 2 (i.e., “PubChem” similarity ≥ 0.60 and “Extended” similarity ≥ 0.30) were considered within the model’s AD.

#### istkNN

*Data split* The same data split described in "BRF/rRF" paragraph ("Data split") was used for selecting model’s optimal parameters (see below). Once optimal parameters were selected, the global TS (iTS + iVS) was used to derive a new model that was validated on the ES.

*Algorithm* istkNN is a commercial tool [38] implementing a modified k-Nearest Neighbors (kNN) algorithm. kNN estimates the outcome of a sample in a dataset on the basis of read-across accounting for the *k* most similar samples (neighbors) in the TS for which the outcomes are known [39, 40]. If the algorithm is applied for predicting continuous endpoints (e.g., LD50 point estimates) the mean of the activities of neighbors is calculated [41].

*Similarity* Similarity between chemicals is described by an integrated similarity index (SI) ranging from 1 (maximum similarity) to 0 (minimum similarity), resulting from a weighted combination of a binary fingerprint array and three non-binary structural keys, as follow:

$${\text{SI}}\, = \,{\text{S}}\left( {\text{FP}} \right)0.4*{\text{S}}\left( {\text{CD}} \right)0.35*{\text{S}}\left( {\text{HE}} \right)0.1*{\text{S}}\left( {\text{FG}} \right)0.15$$

where CD are structural keys with 35 constitutional descriptors (MW, nr of skeleton atoms, etc.), HE are structural keys with 11 hetero-atoms descriptors and FG are structural keys with 154 functional groups (specific chemical moieties) as implemented in Dragon (v. 7.0.8, Kode srl, 2017) (Talete srl, Milano, Italy). FP are the extended fingerprints, which comprise Daylight notation (http://www.daylight.com/dayhtml/doc/theory/theory.finger.html) and additional bits accounting for ring features. FP similarity is calculated with Maxwell-Pilliner index while CD, HE, and FG similarities are calculated with Bray–Curtis index [42].

*Applicability domain* istKNN refines the classical kNN algorithm by setting additional conditions that a sample (i.e. chemical) should fulfill to be considered reliably predicted. The *k* nearest neighbors used for prediction should have a similarity value with the target greater than a given threshold (T_sim1_), otherwise they are not used for prediction. If no neighbors match the threshold, the model does not provide a prediction for the target compound. If only one neighbor matches the threshold, the similarity should be higher than a second stricter threshold (T_sim2_) to return a prediction (which is equal in this case to the experimental values of this selected neighbor). If two or more neighbors fulfill the T_sim1_, the range of experimental values of retained neighbors is considered. If the difference between the maximum and minimum experimental values of neighbors is lower than a threshold (T_min–max_), the target is predicted, otherwise the model does not return predictions. To calculate the prediction when more than one neighbor is selected, the experimental values of the similar compounds can be weighted differently on the basis of their similarity with the target (by setting an enhancement factor that increases the weight to the most similar compounds in the prediction).

In the present work, a batch process was used to optimize the settings of the five customizable parameters according to the following criteria: (a) number of neighbors from 2 to 5; (b) T_sim1_ from 0.70 to 0.90 (step = 0.50); T_sim2_ = 0.85 or 0.90; (c) enhancement factor from 1 to 3; (d) T_min-max_ from 1.0 to 2.0 (step = 0.50).

The iTS was used for the development of (Q)SAR models using all the possible combinations of the parameters above. A restricted pool of valid models was pre-selected based on leave-one-out cross-validation on the iTS, seeking a good compromise in terms of coverage and performance. The final model was the one among the pool with the best performance in external validation on the iVS with a coverage of at least 0.85 (arbitrary threshold). Finally, the selected parameter settings were used to derive a new model on the global TS (iTS + iVS) and to use it for the prediction of the ES.

#### SARpy

*Data split* The entire TS was used to derive SARpy models.

*Algorithm* The freely available SARpy software (https://www.vegahub.eu/portfolio-item/sarpy/) was used to build classification models for nT and vT classifications.

Given a TS of molecular structures described by SMILES, SARpy applies a recursive algorithm considering every combination of bond breakages working directly on the SMILES string. The software generates every structural fragment included in the TS that is then encoded into SMARTS (www.daylight.com/dayhtml/doc/theory/).

Each substructure is validated as a potential structural alert (SA) by verifying the existing correlation between the incidence of a particular molecular fragment and the class of activity of the molecules containing it. In this way, a reduced ruleset of relevant SAs is defined. Each SA was associated with an activity label (e.g., positive or negative) and a likelihood ratio, estimating the statistical relevance of the SA [43, 44].

*Applicability domain* If a chemical does not contain any fragment present in the rule set, it is not predicted and is considered outside of the model AD.

#### Random forest with hyper parameter tuning (HPT-RF)

*Data split* The whole TS was used to derive HPT-RF models.

*Algorithm* “caret” [37] and “ranger” [45] R packages were used to develop regression (LD50) and multi-category classification (EPA and GHS) RF models [28]. Hyperparameter tuning (HPT) research was performed during the RF derivation, in order to select an “optimal” model across various parameters. Selection was made by evaluating the effect of model tuning on performance (i.e., accuracy) in internal validation (i.e., bootstrap). Three parameters were tuned by grid search: (1) *mtry* (number of randomly selected descriptors used in each tree of the RF), (2) *splitrule* (the rule used to choose descriptors for a single tree, i.e. “*gini*” or “*extratrees*” for classification; “*variance*” or “*extratrees*” for regression), (3) *min.node.size* (minimal node size of trees). The number of trees was equal to 500. The reader is referred to the user’s guides of the above-indicated packages for further details.

A first model run served to evaluate the presence of response outliers within the TS. The isolation forest [46] method for anomalies isolation as implemented in the “isofor” R package was used to identify outliers. In addition, chemicals were flagged as outliers if they were characterized by a high variance and high error among iterations of bootstrap internal validation (100 iterations). In a second run, outliers were excluded from model’s derivation.

*Descriptors* Calculation, pruning and normalization of descriptors were the same as in "rRF/BRF" paragraph ("Descriptors").

*Applicability domain* Two approaches were applied for the definition of models AD:

- *Similarity* PubChem Fingerprints (ftp://ftp.ncbi.nlm.nih.gov/pubchem/specifications/pubchem_fingerprints.txt) were calculated for each compound starting from SMILES using the “rcdk” R package. Fingerprint-based similarity (Tanimoto) between a target compound and all the compounds in the TS was computed. If the mean similarity with the three most similar compounds among those flagged as outliers was higher than the similarity with three most similar compound from the TS cleaned from outliers, the compound was considered out of AD.
- *“Dummy” matrix* Descriptors of TS chemicals were randomly permuted (vertical permutation) to create a mirror TS. The shuffled TS was merged with the original one. Samples of the original TS were flagged as “real”, while those of the mirror TS were flagged as “dummy”. A RF classification model was built to distinguish real from dummy samples. External chemicals classified as “dummy” were considered outside of the model’s AD [47, 48].

#### Generalized linear model (GLM)

*Data split* The same data split described in "rRF/BRF" paragraph ("Data split")  was used. The original iTS for the nT classification was further split by 20% of the iTS as an internal calibration set (iCS, 1330 chemicals) in order to evaluate the accuracy of the model during the building process. The iVS was used for validating the final model.

*Algorithm* The model was built using the H_2_O 3.16.0.3 (https://www.h2o.ai/download/) package for GLM in R v. 3.4.0 (https://www.R-project.org).

Logistic regression (LR) is used for binary classification problems when the response is a categorical variable with two levels. In this case, only the nT endpoint was modelled. This approach was not applied to the vT endpoint due to a strong bias arising from few chemicals having a vT designation, and the inadequacy of the method in modeling highly unbalanced datasets. LR-GLM model was fitted by finding a set of parameters that maximizes the probability of an observation belonging to its experimental category. Parameter tuning was performed on the iCS. Penalties were also introduced to the model building process to avoid over-fitting, reducing variance of the prediction error and handling correlated predictors. Penalties (elastic method) were controlled by parameters $\upalpha$ and λ that were set to 0.09 and 0.02, respectively. A more in-depth description of the algorithm is reported by [49].

*Descriptors* Calculation, pruning and normalization of descriptors were performed as in "rRF/BRF" paragraph ("Descriptors").

*Applicability domain* AD was not implemented for this model.

### Integrated modeling

Integrated modeling was applied to all five endpoints. The approach was based on the intuitive assumption that taking into account predictions from multiple models could compensate for the limitations of single models. Different approaches were used for regression and classification endpoints:

- Integrated model for predicting LD50 point estimates was obtained by averaging predictions of the four individual models (i.e., rRF, aiQSAR, istkNN, HPT-RF). Only predictions within the AD of each model were considered for integration. The integrated model’s AD was implemented by applying the concept of “integrated prediction fraction” (PF) described by [50]. In particular, the number of models returning a prediction in AD and contributing to the final integrated prediction for a given sample was considered. The higher was the number of used models, the more reliable the integrated prediction. A threshold was set based on the PF and then integrated predictions with a PF lower than the threshold were considered out of the integrated AD.
- Integrated models for categorical endpoints (vT, nT, EPA, GHS) were obtained by using a majority voting approach. Similarly to above, a consensus score (CS) was used as an index of prediction reliability. In this case, CS was calculated as the number of single models returning the same prediction as the integrated one, minus the number of models returning a prediction different from the integrated one. Only predictions within the AD of each model were considered for integration. For example, three out of four nT models returned predictions for a given sample, two of them being positive and one negative. In this case, the integrated prediction for the sample was “positive” with a CS of 1. No predictions were returned in case of ties.

For all the endpoints, the variation of integrated performance with respect to the PF/CS was evaluated.

## Results

### Model parameterization

Table 3 reports final settings for each model developed with the rRF and BRF method.

For the istkNN approach, the selected model was characterized by the use of 3 neighbors maximum, T_sim1_ = 0.80, T_sim2_ = 0.85, enhancement factor = 3 and T_min-max_ = 2.0.

For the SARpy model, 349 fragments were identified for the vT endpoint (64 for the vT class, 285 for the not vT class), while 446 fragments were identified for the nT endpoint (228 for the nT class, 218 for the class of toxic compounds). The list of fragments encoded as SMARTS are reported in Additional file 3: Table S3a (vT model) and Table S3b (nT model).

Table 4 indicates the values for the three parameters *mtry*, *splitrule* and *min_node_size* of the HPT-RF model.

**Table 3:** rRF and BRF settings

Endpoint	Model	iTS	iVS	#descrs	#trees	k	T_C_	T_D_	T_E_
LD50 point estimate	rRF	5028	1251	1352	150	1	–	90th	1.00
nT	BRF	6722	1680	1247	150	1	0.65	100th	–
vT	BRF	6772	1690	1250	100	1	0.65	95th	–
EPA	BRF	6607	1652	1243	100	1	0.40	100th	–
GHS	BRF	6663	1668	1244	100	5	0.30	90th	–

For each model, the size of the internal training set (iTS) and internal validation set (iVS), the number of descriptors (#descrs), the number of trees (#trees) and the tuned parameters for AD definition are indicated

**Table 4:** HPT-RF settings

Endpoint	*mtry*	*splitrule*	*min_nodee.size*
LD50	748	Extratrees	5
EPA	38	Extratrees	1
GHS	38	Extratrees	1

For each model, the number of descriptors in each tree (mtry), the rule for descriptor selection for single trees (splitrule) and the minimal node size of trees (min.node.size) are indicated

### Statistical performance

External validation performance represents the real proof of the predictive power of (Q)SAR models. In this work, results on the common ES allowed a fair comparison of the models’ predictivity. Table 5 shows the performance of the LD50 continuous models on the ES, while Table 6 shows the performance of classification models.

**Table 5:** External performance of single models for predicting single point logLD50 (mmol/kg)

Model	R^2^	MAE	RMSE	#AD	%AD
rRF	0.590	0.432	0.585	1966	*0.907*
aiQSAR	*0.651*	0.390	*0.541*	1843	0.850
istKNN	0.628	*0.387*	0.545	1917	0.884
HPT-RF	0.620	0.398	*0.541*	1885	0.869

For each model, the R^2^, the mean absolute error (MAE), the root-mean squared error (RMSE), the number (#AD) and the percentage (%AD) of predictions in AD are reported. The best values for each metric are italicized

**Table 6:** External performance of single models for predicting classification endpoints (vT, nT, EPA, GHS)

	Model	SEN	SPE	MCC	BA	#AD	%AD
nT	BRF	*0.830*	*0.848*	*0.674*	*0.839*	2100	0.728
	aiQSAR	0.723	0.829	0.556	0.776	2567	0.890
	SARpy	0.772	0.724	0.492	0.748	2488	0.863
	GLM	0.779	0.650	0.425	0.714	2884	*1.000*
vT	BRF	*0.856*	0.903	0.585	*0.880*	2103	0.728
	aiQSAR	0.682	*0.963*	*0.619*	0.822	2572	0.891
	SARpy	0.710	0.896	0.467	0.803	2613	*0.905*
EPA	BRF	0.614	0.851	0.405	0.733	2301	0.805
	aiQSAR	0.603	0.857	0.450	0.730	2547	*0.891*
	HPT-RF	*0.616*	*0.860*	*0.462*	*0.738*	2180	0.763
GHS	BRF	0.539	0.872	0.342	0.705	1410	0.490
	aiQSAR	0.568	0.895	0.469	0.731	1475	*0.512*
	HPT-RF	*0.569*	*0.897*	*0.476*	*0.733*	1291	0.448

For each model, the sensitivity (SEN), the specificity (SPE), the balanced accuracy (BA), the Matthew’s correlation coefficient (MCC), the number (#AD) and the percentage (%AD) of predictions in AD are reported. For multi-category endpoints (EPA and GHS), SEN and SPE are the average of values computed separately for each class, while BA is the arithmetic mean of the average SEN and SPE. The best values for each metric are italicized

Internal validation is not discussed in the present article. The interested reader is referred to Additional file 1: Tables S4 and S5 for internal performance of each model.

Predictions of all models on the TS and ES are included in Additional file 2: Tables S1 and S2.

All continuous logLD50 (mmol/kg) predictive models showed good predictivity on ES. In particular, aiQSAR, istkNN and HPT-RF models showed a similar behavior with an R^2^ higher than 0.60 and RMSE near 0.54. On this basis, the istkNN method was particularly appealing for its intuitiveness and simplicity, compared with other more computationally-demanding methods. The rRF model was slightly less predictive, but with a higher percentage of predictions in AD (%AD) (i.e., 0.900) compared to other models (Table 5).

As far as classification models, Matthew correlation coefficient (MCC) [51] and balanced accuracy (BA) metrics were used for an overall estimation of classification performance for their ability to deal with unbalanced classes. Cooper statistics [52] were also calculated. For multi-category classifiers (EPA and GHS), the generalized formula of MCC reported by Ballabio et al. [53] was used, while BA was obtained as arithmetic mean of averaged sensitivities and specificities calculated for each separate category.

With respect to binary classification models, all the models showed good predictivity on the ES, with BAs close or higher than 0.80 and MCCs often higher than 0.50 (i.e., BRF and aiQSAR). In both cases, the best techniques was BRF, that returned BAs in external validation of 0.839 (for nT) and 0.880 (for vT), and MCCs of 0.674 (for nT) and 0.585 (for vT), despite a slightly lower %AD (i.e. 0.728) with respect to other methods (Table 6). This confirmed our previous experience [54] on the suitability of this technique to effectively handle highly unbalanced datasets.

As far as multi-categorical endpoints, EPA predictive models showed close performance across algorithms, with MCCs higher than 0.40 and BAs close to 0.73 in all cases. The HPT-RF method showed high performance, but at the cost of a slight loss in coverage with respect of other methods. aiQSAR was close in terms of performance, but with a gain in %AD (i.e., 0.891 compared to 0.763) (Table 6).

For GHS models, aiQSAR and HPT-RF performed better than BRF. However, coverage for GHS models was disappointing, being close to or lower than 0.50 (Table 6). This is reasonable due to the challenging nature of the GHS endpoint, characterized by a high number of (unbalanced) categories (n = 5) (Table 1).

### Integrated models evaluation

**Fig. 2:**
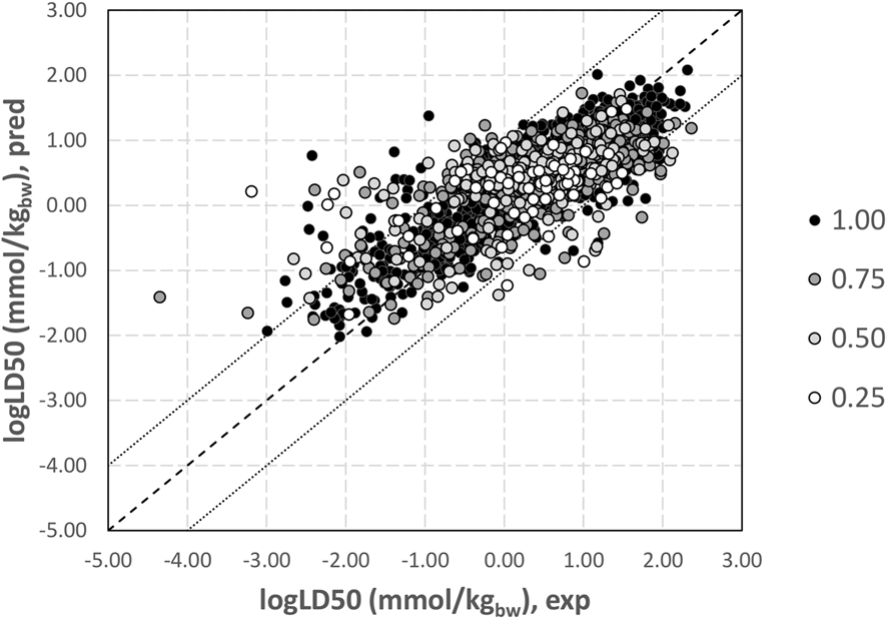
Comparison of experimental and predicted (integrated) logLD50 (mmol/kgbw) for ES chemicals. Darkest circles indicate samples with a high prediction fraction (PF), while lightest circles indicate samples with lower PF. Dashed line represents the case of ideal correlation, while dotted lines delimit samples with an absolute error in prediction lower than 1.00 log unit

Integrated modeling was applied in order to improve predictions of single models. Table 7 shows the performance on ES of integrated models obtained by averaging single LD50 predictions, while Fig. 2 compared the experimental logLD50 (mmol/kg_bw_) values with the predictions returned by the integrated model.

**Table 7:** External performance of the continuous integrated model for predicting single point logLD50 (mmol/kg)

R^2^	MAE	RMSE	#AD	%AD	PF
0.632	0.397	0.549	2152	0.992	0.25
0.646	0.390	0.535	2085	0.961	0.50
0.675	0.373	0.512	1900	0.876	0.75
0.716	0.348	0.477	1474	0.680	1.00

The R^2^, the mean absolute error (MAE), the root-mean squared error (RMSE), the number (#AD) and the percentage (%AD) of predictions in AD are reported, with respect to the PF threshold for defining predictions in AD

Table 8 shows performance on ES of integrated classification models. As expected, the increase of the PF/CS threshold to exclude predictions from the AD always paired with an increased performance, although at the cost of a reduced percentage of predicted compounds. This was especially true for classification when a complete agreement among integrated predictions was required (i.e., maximum CS value) and the number of categories was higher (e.g., GHS integrated models, having %AD = 0.214 for CS = 3). Internal performance on the TS of integrated models is not discussed in the manuscript and is reported in Additional file 1: Tables S6, S7).

**Table 8:** External performance of continuous models for predicting classification endpoints (vT, nT, EPA, GHS)

	SEN	SPE	MCC	BA	#AD	%AD	CS
nT	0.794	0.796	0.587	0.795	2665	0.924	1
	0.840	0.841	0.677	0.840	2182	0.757	2
	0.878	0.858	0.733	0.868	1704	0.591	3
	0.913	0.883	0.794	0.898	1222	0.424	4
vT	0.743	0.938	0.577	0.840	2742	0.949	1
	0.796	0.976	0.737	0.886	2316	0.802	2
	0.890	0.978	0.820	0.934	1556	0.539	3
EPA	0.602	0.856	0.439	0.729	2653	0.928	1
	0.701	0.885	0.550	0.793	1731	0.605	2
	0.739	0.898	0.600	0.819	1200	0.420	3
GHS	0.567	0.894	0.461	0.731	1561	0.542	1
	0.644	0.911	0.541	0.777	908	0.315	2
	0.676	0.916	0.573	0.796	617	0.214	3

For each model, the sensitivity (SEN), the specificity (SPE), the balanced accuracy (BA), the Matthew’s correlation coefficient (MCC) the number (#AD) and the percentage (%AD) of predictions in AD are reported, with respect to the CS threshold for defining predictions in AD. For multi-category endpoints (EPA and GHS), SEN and SPE are the average of sensitivities/specificities computed separately for each class, while BA is the arithmetic mean of the average SEN and SPE

The concept of “Pareto optimum” was applied to retrospectively confirm the improvement of consensus strategy with respect of single models, keeping into account both statistical performance and %AD. The approach allows solving of multi-objective optimization problems, in which no single solution exists that simultaneously optimizes multiple quality criteria. A solution is called “nondominated” (i.e., Pareto optimal) if no other solutions exist for which an evaluation function can be improved without degrading one of the other functions [21, 22].

**Fig. 3:**
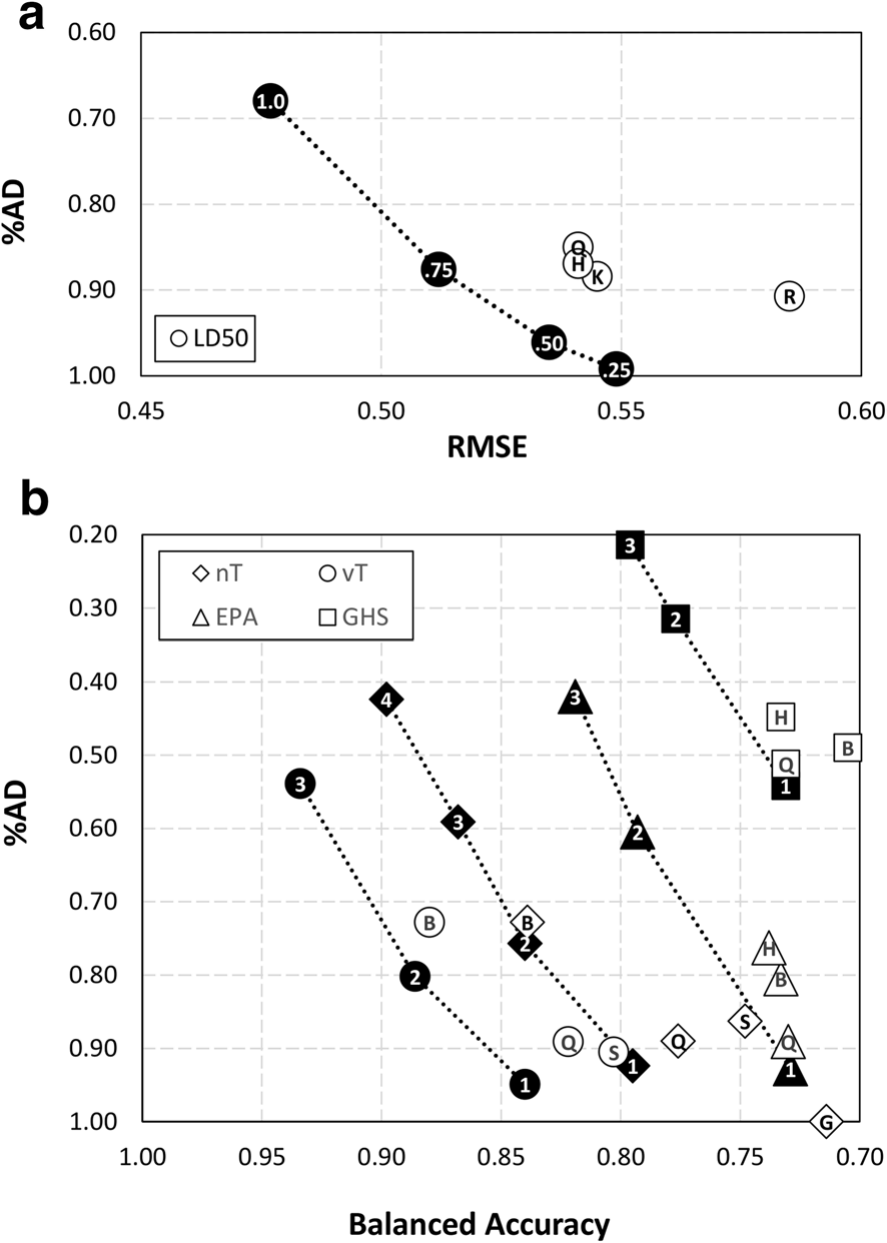
Overview of Pareto optimal solutions for regression and classification models. **a** Performance of continuous LD50 models is described as root-mean squared error (RMSE) versus percentage of compounds in the AD (%AD). **b** Performance of classification (nT, vT, EPA, GHS) models are described as balanced accuracy (BA) versus percentage of compounds in the AD (%AD). All the parameters refer to the ES. Models in the bottom-left part of the plots are characterized by the best compromise in terms of performance and coverage, with dotted lines representing the Pareto front for a given endpoint. White indicators are single models (R = rRF; B = BRF; H = HPT-RF; K = istkNN; S = SARpy; Q = aiQSAR), while black indicators are integrated models, flagged with the corresponding PF (for regression) or CS (for classification) threshold

Quality functions used in this case were performance in external prediction (i.e., RMSE for regression, BA for classification) and %AD on the ES. Figure 3 graphically shows the set of optimal solutions for each endpoint representing the optimal trade-offs between the two functions. Such solutions constitute a Pareto front [21].

As shown in Fig. 3, integrated models always represented the optimal Pareto solutions for each endpoint, confirming the effectiveness of the integrated approach. Conversely, it was never possible to designate the best integrated model among those obtained varying the PF/CS value. Indeed, the increase of the threshold always resulted an increased performance, but at the cost of a systematic reduction of %AD.

For LD50 point estimate models, the integrated model with PF = 0.75 returned a coverage (i.e., 90%) analogous to best individual models, but with a relevant gain in performance (i.e., RMSE = 0.512 with respect of a mean RMSE = 0.542 for the best three individual models) (Tables 5 and 7).

For vT and nT endpoints, the BRF models were the closest to the Pareto front, however both solutions are dominated by the related integrated model with CS = 2.

As for multi-categorical endpoints, the aiQSARs were both close to the Pareto front and in particular to the integrated model with CS = 1. In this case, aiQSAR and integrated models have comparable performance, with a BA close to 0.730 for both endpoints. In both cases, integrated models showed a gain in %AD with respect to aiQSAR models, i.e. 3.7% additional predicted chemicals for the EPA model and 3.0% for GHS.

## Discussion

This work describes the modeling efforts our research group contributed in the development of new (Q)SAR models for predicting five endpoints (one continuous, four classification) related to acute oral toxicity in rats, as a result of our participation in the collaborative project launched by NICEATM and NCCT [19]. This endpoint is of utmost importance to several regulatory frameworks, being currently the basis for the toxicological classification of chemicals [4].

To date, the recent literature reports only a small number of successful attempts in modeling oral rat acute toxicity [3, 9]. The small number of models available can be explained by the nature of the endpoint and the lack of curated datasets prior to this effort. Indeed, compared with other widely modeled endpoints (e.g., (eco)toxicity) the modeling of mammalian toxicity is challenging, representing the sum of a plethora of toxicological mechanisms, each involving different biological pathways and molecular events concurring to the final effect [3, 55].

Individual (Q)SAR methods often showed inherent limitations in developing single models able to handle different mechanism of action, even more in case of a lack of complete understanding of some of the biological mechanisms contributing to the overall effect (e.g., death) [6, 41]. This issue can, however, be compensated with large enough datasets that adequately represent the diversity of the chemical universe.

An additional obstacle is often represented by the lack of reliable toxicity data in terms of quality, source of experimental data and organisms used [56]. The involvement of absorption, distribution, bioaccumulation, metabolism and excretion aspects further contributes to making the whole scenario even more complex and challenging [3, 56].

As a consequence, (Q)SAR models for this endpoint so far have been largely limited to small datasets restricted to a well-defined class of chemicals while global models are few and, often, not satisfactory in terms of predictive power [3, 9].

Despite this, the demand of in silico tools for such complex in vivo endpoints and (Q)SAR models continues to grow, due to objective resource and ethical limitations related to the execution of in vivo tests for a high number of chemicals. In this regard, the need of developing global models addressing a common endpoint such as acute toxicity have arisen as a primary need [3].

Within the project of NICEATM and EPA’s NCCT Acute Toxicity Modeling Consortium, the availability of a large and high-quality rat oral acute toxicity database and the involvement of the entire scientific community represented an unprecedented chance to improve the current state of the art on the in silico modeling of this endpoint. To the best of our knowledge, this is the largest curated dataset of heterogeneous chemicals made available so far for the development of new (Q)SARs.

Integrated modeling was also applied in order to improve predictions of single models. As demonstrated previously and in the current work, integrated models had the highest external prediction power compared to any individual model used in the integrated prediction [57, 58, 59].

This is particularly true when the individual models have been developed using different techniques, have ADs differently defined and showed different behaviors based on the structural and activity profile of predicted chemicals. For example, an inspection of correct predictive rates that classification models have on specific categories of toxicity showed that some methods performed better on certain classes (e.g. more toxic or less toxic compounds) than others (for detailed statistics see Additional file 1: Tables S8–S11).

In this regard, the integrated method can compensate for and correct the limitations of individual techniques, as well as afford greater chemical space coverage [57, 59].

Given the improvement of statistical performance, the use of PF/CS as a way to define AD of integrated models proved to be particularly suitable for identifying predictions likely to be wrong. Another advantage of PF/CS metrics is that these values can be applied to assign a degree of reliability to the prediction. Therefore, this metric can work as an indicator of the likelihood that a compound is within the AD. This meets the need of accounting for the fuzzy nature of boundaries of AD [60], instead of considering the AD assessment as a yes/no issue (e.g., a compound with an intermediate PF value can be considered questionable instead of out of AD). This is confirmed by an inspection of integrated LD50 models’ performance limited to a given PF value. As shown in Table 9, statistical performance on the ES recalculated with respect to a given PF value clearly showed that samples characterized by lower PFs also showed an overall higher error. Looking at external predictions (i.e., ES), samples with PF = 1.00 have the lower RMSE. On the other hand, the value dramatically increases considering samples with lower PFs, to a maximum value of 0.865 in case of PF = 0.25. The effectiveness of the PF/CS approach is further reinforced by looking at the percentage of samples with a high prediction error (i.e., equal or higher than 1.00 log unit) for each PF value, that is about 20% of the total number of samples with PF = 0.25 and only 4.3% for PF = 1.00 (Table 9).

**Table 9:** External performance of the continuous integrated model for separate PFs

R^2^	MAE	RMSE	#	%	%AE ≥ 1	PF
0.265	0.620	0.865	67	0.031	0.209	0.25
0.391	0.568	0.736	185	0.085	0.151	0.50
0.540	0.458	0.616	426	0.196	0.085	0.75
0.716	0.348	0.477	1474	0.680	0.043	1.00

The R2, the mean absolute error (MAE), the root-mean squared error (RMSE), the number (#) and the percentage (%) of predictions with a given prediction fraction (PF) are reported. In addition, the percentage of samples with a given PF value and an absolute error in prediction equal to or greater than 1.00 log unit with respect of the total number of samples with the same PF (%AE ≥ 1) are reported

Machine learning methods we used (e.g., RFs) proved to be valid in terms of predictive performance, but a mechanistic interpretation of these models is often more difficult than classical linear models. Indeed they can be based on thousands of different molecular descriptors and the relationship existing between the endpoint and each descriptor is often a complex, non-linear one that is only implicitly included in the model itself. With this in mind, we provided an analysis of most relevant features used in the descriptor-based global models here presented (rRF/BRF, HPT-RF, GLM). Models generated with aiQSAR and istKNN were not considered in this analysis as they are local models not capable of identifying features associated with the global trend of acute toxicity. In addition, istKNN is based on the similarity concept only and not on descriptors.

The top twenty Dragon descriptors for each of the above-cited models were listed in Additional file 1: Table S12. Details on how the importance of descriptors was determined for each model were included in the same Table. Descriptors belonging to the 2D Atom Pairs category (binary, frequency or weighted topological atom pairs) were the most frequent (79 out of 180 descriptors). They were followed by 2D autocorrelation (16), CATS2D (14), functional group counts (11) and P_VSA-like descriptors (11). P_VSA_s_1 (P_VSA-like on I-state, bin 1) was also the single most frequent descriptor (it was included in four out of nine models considered). P_VSA descriptors are related to van der Waals surface area of chemicals and, indirectly, with their size and lipophilicity. Low LD50 compounds in the TS were characterized by higher values of P_VSA descriptors, suggesting a role of molecular size and lipophilicity in the offset of acute toxicity (e.g., in absorption/excretion and bioaccumulation of chemicals in tissues).

Looking at 2D Atom Pairs descriptors, those referring to the presence of phosphorus (31), carbon (26), fluorine, nitrogen and sulfur (19) atoms were those more frequent. In particular, chemicals having a higher number of phosphorus, fluorine and sulfur atoms had lower LD50 values with respect to the mean of the distribution of values for the TS. The number of phosphorus (nP) was also one of the single most frequent descriptors, that was found in HPT-RF models for EPA and GHS and in BRF/rRF models for single point LD50 and vT classification. Binary/frequency phosphorous-based 2D Autocorrelation descriptors (B01[O-P], B04[C-P] and F02[C-P]) also appeared in multiple models (three models each).

Functional group counts are easier to be interpreted with respect to other theoretical descriptors, because they describe the presence of well-defined structural motif. For example, high values of nOHp (number of primary alcohols) were characteristic of low-toxicity chemicals. High numbers of hydroxyls flagged for high solubility of chemicals that influence the excretion rate, the capability to cross biological membranes and accumulate in tissues to exert toxicity. The presence of primary hydroxyls is important for phase II metabolism (conjugations) that contribute to detoxification of chemicals [17]. On the contrary, high number of aliphatic tertiary alcohols (nOHt) and aliphatic tertiary amines (nRNR2) was observed in high-toxicity compounds. It is possible that high counts of tertiary groups flags for bulky, lipophilic molecules that are easily to accumulate in the organism. Conversely, beta-Lactams, sulphonic and sulphuric acids, that are more hydrophilic, are only presents in safest toxicity categories.

Fragments identified by the SARpy model for vT class with LR = inf were evaluated too. Compared to the functional groups they can include larger fragments. The chemical moieties spotted with these fragments are halogenated 2-trifluoromethyl benzimidazoles, dioxins, phosphonothioates, organothiophosphates (including organothiophosphate aliphatic amides). They refer especially to chemical classes well represented among pesticides or former pesticides active ingredients.

## Conclusions

In the present study, a series of computational models were developed as part of the NICEATM and EPA’s NCCT collaborative project, for the prediction of five regulatory relevant endpoints describing rat acute oral toxicity (LD50). A series of different (Q)SAR methods were applied and the obtained models were validated on a large external dataset to assess their predictivity. Briefly, no single methods proved to be the best for all the endpoints, despite some of them constantly returning highly satisfactory predictive performance. In particular, results showed that some machine learning methods (e.g. RFs) were especially effective in modeling this kind of composite endpoint. These findings support the fact that machine learning approaches have often been indicated as promising tools in the field of computational toxicology [61, 62], and that they are able to handle multiple mechanisms of actions better than classical linear approaches [63, 64].

A review from Gonella-Diaza et al. [15] recently proposed an evaluation of the performance of existing models predicting LD50 implemented in a series of computational platforms. It was shown that only a few models were able to deliver acceptable predictive performance. Indeed, the best models among those evaluated returned RMSE values in external validation and within AD never lower than 0.55 for regression, while accuracy values for the five-class GHS classification ranged between 0.45 and 0.56. The models presented here showed robust performance in external validation, with RMSE values close to 0.50 for integrated models and BAs exceeding 0.80. In this regard, the models presented here can be easily considered an improvement on the current state-of-the-art for in silico modeling of this endpoint.

Another important outcome of this study is that integrated methods always returned improved performance with respect to single models. This confirms, as has already been widely reported, that the integration of multiple strategies and the application of a weight-of-evidence approach solves the limitations inherent to single methods and increases the confidence in the final toxicological prediction.

Several other groups were involved in NICEATM/NCCT collaborative project, and other (Q)SAR models were developed and validated starting from the same data used here. For the ease of example, Alberga et al. [65] developed a multi-fingerprints similarity approach for predicting the five relevant toxicology endpoints related to the acute oral systemic toxicity. The approach integrated the results coming from a similarity search based on 19 different fingerprint definitions to return a consensus prediction value. The algorithm also accounted for toxicity cliffs, i.e. large gaps in LD50 values existing between two highly similar chemicals. Ballabio et al. [55] proposed a Bayesian consensus approach integrating three different mathematical methods for modeling the nT and vT classification endpoints, i.e., N-Nearest Neighbors, Binned Nearest Neighbors and Naïve Bayes classifier. Extended connectivity fingerprints were used to describe the structure of chemicals. Both papers also defined the AD of (Q)SAR models with the aim of identifying the most reliable predictions. External validation results reported by authors were made on the same ES used in this work and were summarized in Table 10.

**Table 10:** External performance of other published acute toxicity models developed within the NICEATM and EPA’s NCCT collaborative project

Method	LD50 single point	nT	vT	EPA	GHS
Multifingerprint similarity search [67]	R2 = 0.737 RMSE = 0.408 %AD = 0.347	SEN = 0.873 SPE = 0.915 MCC = 0.793 %AD = 0.263	SEN = 0.789 SPE = 0.998 MCC = 0.857 %AD = 0.320	MCC = 0.730 %AD = 0.159	MCC = 0.733 %AD = 0.223
Bayesian consensus [55]	–	SEN = 0.800 SPE = 0.840 BA = 0.820 %AD = 0.730	SEN = 0.850 SPE = 0.940 BA = 0.900 %AD = 0.770	–	–

For each method, performance for the five acute toxicity relevant endpoints are reported

Leveraging the collective expertise of the entire scientific community in a collaborative effort was the main aim of the NICEATM/NCCT initiative. Given the encouraging results of this first exercise, as well as the comparable results in validation of other research groups, authors strongly believe that the development of new, comprehensive integrated models will represent a further improvement to the already satisfactory results here presented. Indeed, the combination of several methods will mitigate the weaknesses of single models, towards a better collective consensus approach, as well as an enlarged chemical domain. Finally, all the predictions will be hosted on the EPA’s Chemistry Dashboard and made freely available to the entire scientific community.

## Supplementary information

**Additional file 1.** Tables S4–S12 reporting additionals statistics for models, and details on dataset curation are included.

**Additional file 2.** Tables S1 and S2 including experimental values and predictions of models for TS and ES are included.

**Additional file 3.** Tables S3a and S3b with lists of SMARTS implemented in SARpy are included.

**Additional file 4.** The list of descriptors used for models derivation are included.

## Data Availability

The datasets supporting the conclusions of this article are included within the article and in Additional files 1, 2, 3 and 4.

## Abbreviations

aiQSAR: ab initio QSAR
AD: applicability domain’s
ADM: applicability domain measure
BA: balanced accuracy
BRF: balanced random forest
CS: consensus score
ES: evaluation set
EPA: Environmental Protection Agency
GHS: Globally Harmonized System of Classification and Labelling
iCS: internal calibration set
iTS: internal training set
iVS: internal validation set
kNN: k-Nearest Neighbors
MCC: Matthew’s correlation coefficient
LD50: median lethal dose
NCCT: National Center for Computational Toxicology
nT: Nontoxic
NICEATM: NTP Interagency Center for the Evaluation of Alternative Toxicological Methods
%AD: percentage of predictions in AD
PF: prediction fraction
(Q)SAR: (quantitative) structure activity relationship
HPT-RF: random forest with hyperparameter tuning
rRF: random forest in regression
RMSE: root-mean squared error
SA: structural alert
TS: training set
vT: very toxic
