# Supplementary material for: SAR and QSAR modeling of a large collection of LD50 rat acute oral toxicity data
Source: J Cheminform. 2019 Aug 30;11:58. doi: 10.1186/s13321-019-0383-2 (PMC6717335; doi:10.1186/s13321-019-0383-2)
Supplement: Supplementary file 1 — Additional file 1. Tables S4–S12 reporting additionals statistics for models, and details on dataset curation are included. [file 13321_2019_383_MOESM1_ESM.docx]

**Additional file 1**

**SAR AND QSAR MODELLING OF A LARGE COLLECTION OF LD_50_ RAT ACUTE ORAL TOXICITY DATA**

Domenico Gadaleta^1*^, Kristijan Vukovic^1^, Cosimo Toma^1,2^, Giovanna J. Lavado^1^, Agnes L. Karmaus^3^, Kamel Mansouri^3^, Nicole Kleinstreuer^4^, Emilio Benfenati^1^ and Alessandra Roncaglioni^1^

*^1^Laboratory of Environmental Chemistry and Toxicology, Department of Environmental Health Sciences, IRCCS - Istituto di Ricerche Farmacologiche Mario Negri, Via Mario Negri 2, 20156 Milan, Italy*

*^2^Institute for Risk Assessment Sciences, Utrecht University, PO Box 80177, 3508 TD Utrecht, The Netherlands*

*^3^Integrated Laboratory Systems, Inc., Research Triangle Park, NC 27560, USA*

*^4^NTP Interagency Center for the Evaluation of Alternative Toxicological Methods, National Institute of Environmental Health Sciences, Research Triangle Park, NC 27560, USA*

** Author for correspondence.*

*Email address: domenico.gadaleta@marionegri.it*

***METHODS***

***Dataset curation - LD50 single estimates***

For the compilation of the continuous LD50 point estimates TS used in this work, source data were retrieved from the “Complete LD50 Inventory” (https://ntp.niehs.nih.gov/iccvam/methods/acutetox/model/trainingset_full_ld50.txt). This support file has a dataset comprising 8,994 CASRN and 12,184 LD50 values (point estimates and limit tests), as opposed to the training set file provided by organizers in which they applied a workflow to identify a single representative value per CASRN. The support file, “Complete LD50 Inventory” contained some chemicals with multiple LD50 values, as identified during the compilation of the data, which we processed independently to compute our own representative values per chemical.

First, we retrieved QSAR-ready structures in the form of SMILES and InChI unique identifiers that were provided in the training set document by the organizers (i.e., TrainingSet.txt; https://ntp.niehs.nih.gov/iccvam/methods/acutetox/model/trainingset_171130.txt), and associated to each record.

LD50 single point values (provided as mg/kg_bw_ in the original documents) were converted to logLD50 (mmol/kg_bw_) to have a distribution of data more suitable for modelling. The molecular weights used for conversion were calculated as the sum of the molecular weights of the main molecules and of their counterions, if present.

All the records from the “Complete LD50 inventory” referring to the same chemical (same InChI code) were put together and aggregated. Counterions were removed and not considered during the aggregation. Only “point estimate” values were considered while “limit tests” were rejected.

For each chemical, the median of the first quartile and the standard deviation of the distribution of aggregated values was calculated. The median of the first quartile was used as continuous endpoint value for LD50 single point estimates modeling. Chemicals having standard deviation ≥ 0.50 log units among experimental logLD50 (mmol/kg_bw_) values were excluded only for modelling of LD50 single point estimates, eliminating 89 chemicals.

Analogously to the TS, LD50 single point estimates values (mg/kg) in the ES were converted to logLD50 (mmol/kg) to compare experimental values to predictions generated by the models.

***Data curation - Classification endpoint***

For the compilation of the four classification TSs (i.e., vT, nT, EPA and GHS endpoints) used in this work, source data were retrieved from the TrainingSet.txt file (https://ntp.niehs.nih.gov/iccvam/methods/acutetox/model/trainingset_171130.txt) providing CASRN, QSAR-ready structures in the form of SMILES, and a single value per chemical corresponding to each of the endpoints.

All the records referring to the same chemical (same InChI code) were put together and aggregated, while counterions were ignored. If a single chemical resulted from the aggregation of multiple records having different categories for a classification endpoint, they were excluded from the final dataset for that endpoint because it was impossible to assign them to a unique category; this process eliminated 11 chemicals from the TS for vT endpoint, 54 chemicals for nT, 103 chemicals for EPA, and 96 chemicals for GHS categorization.

**TABLES**

**Table S4. Internal performance of single models for predicting single point logLD50 (mmol/kg).** For each model, the R^2^, the mean absolute error (MAE), the root-mean squared error (RMSE), the number (#AD) and the percentage (%AD) of predictions in AD are reported. Performance of the rRF model are the combination of cross validation (CV) predictions on the iTS and external performance on the iVS.

| **Model** | **Validation** | **R^2^** | **MAE** | **RMSE** | **#AD** | **%AD** |
| --- | --- | --- | --- | --- | --- | --- |
| rRF | iTS (10-fold-CV)+iVS | 0.577 | 0.434 | 0.586 | 5651 | 0.900 |
| aiQSAR |  | 0.602 | 0.406 | 0.573 | 5335 | 0.850 |
| istKNN | TS | 0.600 | 0.410 | 0.575 | 5534 | 0.881 |
| HPT-RF | TS (bootstrap) | 0.621 | 0.390 | 0.511 | 5976 | 0.952 |

**Table S5. Internal performance of single models for predicting classification endpoints (vT, nT, EPA, GHS).** For each model, the sensitivity (SEN), the specificity (SPE), the Balanced Accuracy (BA), the Matthew’s Correlation Coefficient (MCC), the number (#AD) and the percentage (%AD) of predictions in AD are reported. For multi-category endpoints (EPA and GHS), SEN and SPE are the average of values computed separately for each class, while BA is the arithmetic mean of the average SEN and SPE. Performance of the BRF model are the combination of cross validation (CV) predictions on the iTS and external performance on the iVS.

|  | **Model** | **Validation** | **SEN** | **SPE** | **MCC** | **BA** | **#AD** | **%AD** |
| --- | --- | --- | --- | --- | --- | --- | --- | --- |
| **nT** | BRF | iTS (10-fold-CV)+iVS | 0.820 | 0.828 | 0.645 | 0.824 | 6001 | 0.714 |
|  | aiQSAR |  | 0.745 | 0.822 | 0.568 | 0.783 | 7419 | 0.883 |
|  | SARpy | TS | 0.840 | 0.775 | 0.611 | 0.808 | 7310 | 0.870 |
|  | GLM | iTS+iCS+iVS | 0.814 | 0.670 | 0.480 | 0.742 | 8402 | 1.000 |
| **vT** | BRF | iTS (10-fold-CV)+iVS | 0.855 | 0.910 | 0.598 | 0.883 | 5922 | 0.700 |
|  | aiQSAR |  | 0.665 | 0.963 | 0.606 | 0.814 | 7475 | 0.883 |
|  | SARpy | TS | 0.836 | 0.900 | 0.553 | 0.868 | 7782 | 0.920 |
| **EPA** | BRF | iTS (10-fold-CV)+iVS | 0.630 | 0.852 | 0.411 | 0.741 | 6505 | 0.788 |
|  | aiQSAR |  | 0.600 | 0.857 | 0.448 | 0.728 | 7289 | 0.883 |
|  | HPT-RF | TS (bootstrap) | 0.592 | 0.856 | 0.445 | 0.724 | 6722 | 0.814 |
| **GHS** | BRF | iTS (10-fold-CV)+iVS | 0.513 | 0.872 | 0.337 | 0.693 | 4212 | 0.506 |
|  | aiQSAR |  | 0.545 | 0.894 | 0.462 | 0.720 | 4124 | 0.495 |
|  | HPT-RF | TS (bootstrap) | 0.536 | 0.893 | 0.457 | 0.715 | 3878 | 0.465 |

**Table S6. Internal performance of the continuous integrated model for predicting single point logLD50 (mmol/kg).** The R^2^, the mean absolute error (MAE), the root-mean squared error (RMSE), the number (#AD) and the percentage (%AD) of predictions in AD are reported, with respect of the PF threshold for defining predictions in AD.

| **R^2^** | **MAE** | **RMSE** | **#AD** | **%AD** | **PF** |
| --- | --- | --- | --- | --- | --- |
| 0.608 | 0.407 | 0.563 | 6261 | 0.997 | 0.25 |
| 0.625 | 0.398 | 0.548 | 6060 | 0.965 | 0.50 |
| 0.654 | 0.381 | 0.518 | 5650 | 0.900 | 0.75 |
| 0.702 | 0.344 | 0.456 | 4525 | 0.721 | 1.00 |

**Table S7. Internal performance of integrated classification models for predicting classification endpoints (vT, nT, EPA, GHS).** For each model, the sensitivity (SEN), the specificity (SPE), the Balanced Accuracy (BA), the Matthew’s Correlation Coefficient (MCC) the number (#AD) and the percentage (%AD) of predictions in AD are reported, with respect of the CS threshold for defining predictions in AD. For multi-category endpoints (EPA and GHS), SEN and SPE are the average of sensitivities computed separately for each class, while BA is the arithmetic mean of the average SEN and SPE.

|  | **SEN** | **SPE** | **MCC** | **BA** | **#AD** | **%AD** | **CS** |
| --- | --- | --- | --- | --- | --- | --- | --- |
| **nT** | 0.828 | 0.809 | 0.632 | 0.818 | 7820 | 0.931 | 1 |
|  | 0.866 | 0.849 | 0.712 | 0.858 | 6427 | 0.765 | 2 |
|  | 0.916 | 0.869 | 0.782 | 0.892 | 5060 | 0.602 | 3 |
|  | 0.953 | 0.890 | 0.841 | 0.921 | 3583 | 0.426 | 4 |
| **vT** | 0.802 | 0.945 | 0.634 | 0.874 | 8015 | 0.947 | 1 |
|  | 0.861 | 0.976 | 0.777 | 0.919 | 6721 | 0.794 | 2 |
|  | 0.919 | 0.975 | 0.823 | 0.947 | 4489 | 0.530 | 3 |
| **EPA** | 0.598 | 0.855 | 0.436 | 0.727 | 7759 | 0.939 | 1 |
|  | 0.689 | 0.882 | 0.540 | 0.786 | 4967 | 0.601 | 2 |
|  | 0.748 | 0.903 | 0.615 | 0.825 | 3340 | 0.404 | 3 |
| **GHS** | 0.540 | 0.891 | 0.443 | 0.715 | 4480 | 0.538 | 1 |
|  | 0.604 | 0.908 | 0.528 | 0.756 | 2493 | 0.299 | 2 |
|  | 0.642 | 0.918 | 0.579 | 0.780 | 1807 | 0.217 | 3 |

**Table S8. Internal validation class-specific predictive rates of single models for predicting classification endpoints (vT, nT, EPA, GHS).** Predictive rates are the percentage number of correctly predicted compounds belonging to a given experimental class. For the vT classification, class 1 corresponds to the positive (i.e., very toxic) compounds, while for the nT endpoint class 1 corresponds to the negative (i.e., toxic) compounds. For each model, the number (#AD) and the percentage (%AD) of predictions in AD are reported.

|  | **Model** | **Validation** | **Class 1** | **Class 2** | **Class 3** | **Class 4** | **Class 5** | **#AD** | **%AD** |
| --- | --- | --- | --- | --- | --- | --- | --- | --- | --- |
| **nT** | BRF | iTS (10-fold-CV)+iVS | 0.828 | 0.820 | - | - | - | 6001 | 0.714 |
|  | aiQSAR |  | 0.821 | 0.745 | - | - | - | 7419 | 0.883 |
|  | SARpy | TS | 0.775 | 0.840 | - | - | - | 7310 | 0.870 |
|  | GLM | iTS+iCS+iVS | 0.669 | 0.814 | - | - | - | 8402 | 1.000 |
| **vT** | BRF | iTS (10-fold-CV)+iVS | 0.855 | 0.910 | - | - | - | 5922 | 0.700 |
|  | aiQSAR |  | 0.665 | 0.963 | - | - | - | 7475 | 0.883 |
|  | SARpy | TS | 0.836 | 0.900 | - | - | - | 7782 | 0.920 |
| **GHS** | BRF | iTS (10-fold-CV)+iVS | 0.692 | 0.398 | 0.372 | 0.478 | 0.627 | 6505 | 0.788 |
|  | aiQSAR |  | 0.43 | 0.484 | 0.434 | 0.631 | 0.748 | 7289 | 0.883 |
|  | HPT-RF | TS (bootstrap) | 0.395 | 0.471 | 0.44 | 0.636 | 0.74 | 6722 | 0.814 |
| **EPA** | BRF | iTS (10-fold-CV)+iVS | 0.782 | 0.555 | 0.45 | 0.732 | - | 4212 | 0.506 |
|  | aiQSAR |  | 0.604 | 0.558 | 0.759 | 0.477 | - | 4124 | 0.495 |
|  | HPT-RF | TS (bootstrap) | 0.577 | 0.562 | 0.77 | 0.458 | - | 3878 | 0.465 |

**Table S9. External validation class-specific predictive rates of single models for predicting classification endpoints (vT, nT, EPA, GHS).** Predictive rates are the percentage number of correctly predicted compounds belonging to a given experimental class. For the vT classification, class 1 corresponds to the positive (i.e., very toxic) compounds, while for the nT endpoint class 1 corresponds to the negative (i.e., toxic) compounds. For each model, the number (#AD) and the percentage (%AD) of predictions in AD are reported.

|  | **Model** | **Class 1** | **Class 2** | **Class 3** | **Class 4** | **Class 5** | **#AD** | **%AD** |
| --- | --- | --- | --- | --- | --- | --- | --- | --- |
| **nT** | BRF | 0.848 | 0.829 | - | - | - | 2100 | 0.728 |
|  | aiQSAR | 0.829 | 0.723 | - | - | - | 2567 | 0.890 |
|  | SARpy | 0.724 | 0.772 | - | - | - | 2488 | 0.863 |
|  | GLM | 0.649 | 0.779 | - | - | - | 2884 | 1.000 |
| **vT** | BRF | 0.856 | 0.903 | - | - | - | 2103 | 0.728 |
|  | aiQSAR | 0.682 | 0.963 | - | - | - | 2572 | 0.891 |
|  | SARpy | 0.71 | 0.896 | - | - | - | 2613 | 0.905 |
| **GHS** | BRF | 0.76 | 0.43 | 0.401 | 0.486 | 0.619 | 2301 | 0.805 |
|  | aiQSAR | 0.481 | 0.537 | 0.439 | 0.644 | 0.735 | 2547 | 0.891 |
|  | HPT-RF | 0.48 | 0.519 | 0.455 | 0.647 | 0.742 | 2180 | 0.763 |
| **EPA** | BRF | 0.735 | 0.552 | 0.477 | 0.693 | - | 1410 | 0.490 |
|  | aiQSAR | 0.623 | 0.539 | 0.766 | 0.482 | - | 1475 | 0.512 |
|  | HPT-RF | 0.657 | 0.564 | 0.766 | 0.476 | - | 1291 | 0.448 |

**Table S10. Internal validation class-specific predictive rates of integrated models for predicting classification endpoints (vT, nT, EPA, GHS).** Predictive rates are the percentage number of correctly predicted compounds belonging to a given experimental class. For the vT classification, class 1 corresponds to the positive (i.e., very toxic) compounds, while for the nT endpoint class 1 corresponds to the negative (i.e., toxic) compounds. For each model, the number (#AD) and the percentage (%AD) of predictions in AD are reported.

|  | **Class 1** | **Class 2** | **Class 3** | **Class 4** | **Class 5** | **#AD** | **%AD** | **CS** |
| --- | --- | --- | --- | --- | --- | --- | --- | --- |
| **nT** | 0.809 | 0.828 | - | - | - | 0.809 | 0.931 | 1 |
|  | 0.849 | 0.866 | - | - | - | 0.849 | 0.765 | 2 |
|  | 0.869 | 0.916 | - | - | - | 0.869 | 0.602 | 3 |
|  | 0.89 | 0.953 | - | - | - | 0.89 | 0.426 | 4 |
| **vT** | 0.945 | 0.802 | - | - | - | 0.945 | 0.947 | 1 |
|  | 0.976 | 0.861 | - | - | - | 0.976 | 0.794 | 2 |
|  | 0.975 | 0.919 | - | - | - | 0.975 | 0.530 | 3 |
| **GHS** | 0.459 | 0.461 | 0.442 | 0.602 | 0.735 | 0.459 | 0.939 | 1 |
|  | 0.583 | 0.511 | 0.454 | 0.675 | 0.795 | 0.583 | 0.601 | 2 |
|  | 0.625 | 0.571 | 0.481 | 0.707 | 0.829 | 0.625 | 0.404 | 3 |
| **EPA** | 0.614 | 0.554 | 0.724 | 0.502 | - | 0.614 | 0.538 | 1 |
|  | 0.76 | 0.617 | 0.737 | 0.643 | - | 0.76 | 0.299 | 2 |
|  | 0.837 | 0.679 | 0.746 | 0.729 | - | 0.837 | 0.217 | 3 |

**Table S11. External validation class-specific predictive rates of integrated models for predicting classification endpoints (vT, nT, EPA, GHS).** Predictive rates are the percentage number of correctly predicted compounds belonging to a given experimental class. For the vT classification, class 1 corresponds to the positive (i.e., very toxic) compounds, while for the nT endpoint class 1 corresponds to the negative (i.e., toxic) compounds. For each model, the number (#AD) and the percentage (%AD) of predictions in AD are reported.

|  | **Class 1** | **Class 2** | **Class 3** | **Class 4** | **Class 5** | **#AD** | **%AD** | **CS** |
| --- | --- | --- | --- | --- | --- | --- | --- | --- |
| **nT** | 0.796 | 0.794 | - | - | - | 2665 | 0.924 | 1 |
|  | 0.841 | 0.84 | - | - | - | 2182 | 0.757 | 2 |
|  | 0.858 | 0.878 | - | - | - | 1704 | 0.591 | 3 |
|  | 0.883 | 0.913 | - | - | - | 1222 | 0.424 | 4 |
| **vT** | 0.938 | 0.743 | - | - | - | 2742 | 0.949 | 1 |
|  | 0.976 | 0.796 | - | - | - | 2316 | 0.802 | 2 |
|  | 0.978 | 0.89 | - | - | - | 1556 | 0.539 | 3 |
| **GHS** | 0.509 | 0.521 | 0.449 | 0.632 | 0.723 | 2653 | 0.928 | 1 |
|  | 0.714 | 0.551 | 0.49 | 0.675 | 0.789 | 1731 | 0.605 | 2 |
|  | 0.75 | 0.627 | 0.503 | 0.669 | 0.829 | 1200 | 0.420 | 3 |
| **EPA** | 0.632 | 0.542 | 0.73 | 0.503 | - | 1561 | 0.542 | 1 |
|  | 0.808 | 0.612 | 0.743 | 0.641 | - | 908 | 0.315 | 2 |
|  | 0.852 | 0.663 | 0.728 | 0.713 | - | 617 | 0.214 | 3 |

**Table S12. Top descriptors for rRF/BRF, HPT-RF, GLM and models.** Details on how variable ranking was determined for each model were reported in footnotes.

| **#** | **HPT - LD50^1^** | **HPT - EPA^1^** | **HPT - GHS^1^** | **GLM – nT^2^** | **rRF - LD50^3^** | **BRF - vT^3^** | **BRF - nT^3^** | **BRF - GHS^3^** | **BRF - EPA^3^** |
| --- | --- | --- | --- | --- | --- | --- | --- | --- | --- |
| 1 | F02[C-P] | B01[O-P] | F02[C-P] | GATS1m | B01[S-P] | F03[N-F] | N-078 | B02[P-Cl] | B07[F-Br] |
| 2 | ZM1Mad | B03[C-P] | nN | B04[C-N] | B05[C-P] | T(F..Br) | CATS2D_07_NN | nBeta-Lactams | T(F..Br) |
| 3 | SM15_EA(dm) | B04[C-P] | SpPosA_B(m) | CATS2D_01_DN | SM1_Dz(Z) | MATS1m | F01[S-P] | nSO2OH | B06[P-Br] |
| 4 | B01[S-P] | B02[C-N] | GATS6i | F04[C-S] | AVS_B(m) | F05[C-F] | CATS2D_03_PN | nSO3OH | CATS2D_03_PN |
| 5 | SM6_B(m) | B05[C-P] | O-058 | T(N..Cl) | X3v | B06[F-Cl] | B06[F-Br] | T(S..I) | B06[F-Br] |
| 6 | ATS2m | B01[C-N] | P_VSA_e_5 | CATS2D_02_DA | ATS4m | B07[F-Cl] | CATS2D_06_PN | B05[S-Br] | B06[N-I] |
| 7 | SpMax1_Bh(m) | B03[C-N] | SpMAD_AEA(dm) | Uc | ZM1Mad | B02[O-S] | CATS2D_01_DA | F02[F-Cl] | B07[F-Cl] |
| 8 | SpMax2_Bh(m) | nP | nP | B04[C-P] | nHM | nP | CATS2D_02_NN | B01[N-Cl] | B06[F-Cl] |
| 9 | F01[C-N] | B02[O-S] | MAXDN | SpMax2_Bh(v) | P_VSA_MR_8 | T(O..P) | F04[C-Br] | nOHt | T(S..P) |
| 10 | SpMAD_B(m) | F02[C-P] | GATS3i | GATS1p | SM2_B(m) | P_VSA_s_1 | F09[C-Br] | F09[F-F] | B09[O-I] |
| 11 | GATS3i | MATS1m | Eig01_AEA(dm) | P_VSA_s_1 | Mp | nRCNO | F03[O-S] | nArCONH2 | P_VSA_s_1 |
| 12 | nN | B04[C-N] | Hy | nOHp | nP | B04[C-P] | Uc | F06[O-F] | B10[S-S] |
| 13 | ZM2Mad | SM15_EA(dm) | SM15_EA(dm) | nRNR2 | P_VSA_i_1 | B04[N-Br] | CATS2D_02_AA | B09[F-Cl] | F06[Br-Br] |
| 14 | GATS1m | IC0 | B01[O-P] | DLS_05 | B02[O-S] | F01[S-P] | F05[C-S] | B02[N-Si] | CATS2D_08_NN |
| 15 | SssssC | GATS6i | P_VSA_e_3 | nRCONHR | P_VSA_LogP_8 | CATS2D_09_DL | SdsN | B04[P-P] | MATS1m |
| 16 | F05[C-N] | CIC1 | Eig02_AEA(dm) | MATS1e | P_VSA_s_1 | T(N..P) | CATS2D_04_NL | F07[O-Br] | B04[N-Br] |
| 17 | F04[C-N] | GATS1m | SpMAD_B(m) | H-047 | F05[C-P] | B02[P-P] | F06[N-S] | F10[O-F] | CATS2D_03_NN |
| 18 | P_VSA_e_3 | GATS3i | SpMax1_Bh(s) | B01[C-N] | SM6_B(p) | F03[C-P] | F07[O-S] | CATS2D_01_DN | F05[O-F] |
| 19 | C-005 | P_VSA_LogP_4 | F01[C-N] | C-026 | SpMax_B(p) | B03[S-P] | B06[P-F] | nTriazoles | F03[P-Cl] |
| 20 | B01[O-P] | SpMax3_Bh(s) | SpMax1_Bh(m) | GATS1e | SM04_EA(ed) | T(S..P) | D/Dtr08 | nNq | F06[O-Br] |

^1^The variable importance was computed using the option importance= 'impurity' as implemented in the “caret” R package. The ’impurity’ measure is the Gini index for classification and the variance of the responses for regression.

^2^The variable importance was determined looking at absolute values of standardized coefficients assigned to each descriptor in GLM model’s equation.

^3^The variable importance was determined by calculating a score for each attributes, by counting how many times it was selected for a split and at which rank (level) among all available attributes (candidates) in the trees of the ensemble.

Score = #splits(lev.0)/#candidates(lev.0) + #splits(lev.1)/#candidates(lev.1) + #splits(lev.2)/#candidates(lev.2).
